# Germ cell-specific deletion of *Pex3* reveals essential roles of PEX3-dependent peroxisomes in spermiogenesis

**DOI:** 10.7555/JBR.37.20230055

**Published:** 2023-12-08

**Authors:** Yejin Yao, Baolu Shi, Xiangzheng Zhang, Xin Wang, Shuangyue Li, Ying Yao, Yueshuai Guo, Dingdong Chen, Bing Wang, Yan Yuan, Jiahao Sha, Xuejiang Guo

**Affiliations:** 1 State Key Laboratory of Reproductive Medicine and Offspring Health, Nanjing Medical University, Nanjing, Jiangsu 211166, China; 2 Reproductive and Genetic Branch, the First Affiliated Hospital of USTC, Division of Life Sciences and Medicine, University of Science and Technology of China, Hefei, Anhui 230001, China; 3 State Key Laboratory of Reproductive Medicine and Offspring Health, Women's Hospital of Nanjing Medical University, Nanjing Maternity and Child Health Care Hospital, Nanjing Medical University, Nanjing, Jiangsu 211166, China

**Keywords:** male infertility, spermiogenesis, peroxisome, oxidative stress, PEX3

## Abstract

Peroxisomes are organelles enclosed by a single membrane and are present in various species. The abruption of peroxisomes is correlated with peroxisome biogenesis disorders and single peroxisomal enzyme deficiencies that induce diverse diseases in different organs. However, little is known about the protein compositions and corresponding roles of heterogeneous peroxisomes in various organs. Through transcriptomic and proteomic analyses, we observed heterogenous peroxisomal components among different organs, as well as between testicular somatic cells and different developmental stages of germ cells. As *Pex3* is expressed in both germ cells and Sertoli cells, we generated *Pex3* germ cell- and Sertoli cell-specific knockout mice. While *Pex3* deletion in Sertoli cells did not affect spermatogenesis, the deletion in germ cells resulted in male sterility, manifested as the destruction of intercellular bridges between spermatids and the formation of multinucleated giant cells. Proteomic analysis of the *Pex3*-deleted spermatids revealed defective expressions of peroxisomal proteins and spermiogenesis-related proteins. These findings provide new insights that PEX3-dependent peroxisomes are essential for germ cells undergoing spermiogenesis, but not for Sertoli cells.

## Introduction

Peroxisomes are ubiquitous organelles found in virtually all eukaryotic cells. They were first discovered as microbodies and the related particles in mouse kidneys by electron microscopy in 1969^[1–2]^. Various morphological subtypes of peroxisomes with distinct functions have been identified in rat liver and kidney^[2–5]^. Peroxisomes in rat brain are typically smaller and less dense, compared with those found in the livers or kidneys^[3]^. Studies examining the proteome of peroxisomes in mouse and rat livers or kidneys have identified diverse protein compositions, suggesting that peroxisomes play heterogeneous roles in different organs^[6–7]^. Additionally, the visualization of individual peroxisomal proteins has identified different locations of these proteins within the testis^[8]^.

In mammalian cells, the *de novo* peroxisome biogenesis begins with a fusion between mitochondria-derived peroxisomal biogenesis factor 3 (PEX3) containing pre-peroxisomal vesicles and peroxisomal biogenesis factor 16 (PEX16) containing vesicles emerging from the endoplasmic reticulum^[9]^. PEX3 and PEX16 function as import receptors for peroxisomal membrane proteins (PMPs) like ATP binding cassette subfamily D members (ABCDs), which are incorporated into the lipid bilayer with the aid of their cytoplasmic chaperone, the peroxisomal biogenesis factor 19 (PEX19)^[2]^. Subsequently, matrix enzymes, such as glycerophosphate O-acyltransferase (GNPAT), are transported into peroxisomes *via* the PMPs, peroxisomal biogenesis factor 13 (PEX13), and peroxisomal biogenesis factor 14 (PEX14), resulting in the formation of intact functional peroxisomes^[10]^. These organelles are responsible for various metabolic pathways, including scavenging of reactive oxygen species (ROS), synthesis of ether lipids and β-oxidation of very-long-chain fatty acids (VLCFA)^[11]^.

Functional peroxisomes in the testis are essential for male fertility. Disruption of the *Pex* genes blocks peroxisome biogenesis, which is closely linked to severe reproductive defects in mice. For example, global knockout of *Pex3* and germ cell-specific knockout of *Pex13* arrested sperm production at the spermatid stage with the formation of multinucleated giant cells (MGCs) and loss of mature spermatids^[12–13]^; Sertoli cell-specific silencing of *Pex5* decreased the number of spermatozoa in the lumen of seminiferous tubules and increased the formation of large lipid droplets in Sertoli cells^[14]^; mice with a mutation in peroxisomal biogenesis factor 7 (*Pex7*) exhibited testicular atrophy primarily characterized by the devoid of spermatogonia and spermatocytes^[15]^. In the *Gnpat* deleted mice, plasmalogen levels were remarkably decreased with the formation of MGCs in seminiferous tubules, resulting in male sterility^[16]^. Similarly, patients with X-linked adrenoleukodystrophy, who suffered from a defect of ABCD1, exhibited impairment of spermatogenesis^[17]^. These mutations interrupt the peroxisomal matrix import pathway and generate peroxisomal ghosts, thus impairing peroxisomal functions and cause male infertility^[16]^. Despite the functions of some peroxisomal proteins being studied, there remains a lack of systemic analysis of the diverse compositions of heterogeneous peroxisomes, especially in different tissues and testicular cells.

Here, we report a study to compare expressions of peroxisomal proteins between germ cells and somatic cells, particularly in the different stages of germ cells and determine whether PEX3-dependent peroxisomes are essential for sperm formation and male fertility in germ cells or in Sertoli cells.

## Materials and methods

### Animals

All animal experiments were approved by the Institutional Animal Care and Use Committees of Nanjing Medical University, Nanjing, Jiangsu, China (IACUC2009002). *Pex3*^*tm1a(EUCOMM)Wtsi/+*^ mice were obtained from the European Conditional Mouse Mutagenesis (EUCOMM) consortium.* Stra8*-*Cre* mice were purchased from the Jackson Laboratory (Bar Harbor, Maine, USA)^[18]^. *Amh*-*Cre* was generated by Dr. Florian Guillou's lab^[19]^ and was transferred from Dr. Xingxu Huang's lab. All mice were housed in a specific pathogen-free animal facility with a 12-hour light/dark cycle. Mice were genotyped by PCR analysis of the genomic DNA. The wild type (WT) (461 bp) and* Pex3* floxed alleles (265 bp) were assayed by PCR with primers 5′-GCCAAACCATAGCACCAGC-3′ and 5′-CTTTGTCCTCTTTCTGGGCAC-3′. The *Stra8-Cre* allele (179 bp) was assayed by PCR with primers 5′-GTGCAAGCTGAACAACAGGA-3′ and 5′-AGGGA CACAGCATTGGAGTC-3′. The *Amh*-*Cre* allele (481 bp) was assayed by PCR with primers 5′-GCCTGCATTACCGGTCGATGC-3′ and 5′-CAGGGTGTTATAAGCAATCCC-3′.

### Fertility assessment

Ten 8-week-old male mice (*Pex3* conditional knockout) were mated with different WT females (1 : 2 ratio) on four cycles to determine their reproductive capacity. Females were checked for vaginal plugs daily and the number of pups was counted within a week of birth.

### Isolation of spermatogenic cells

Pachytene spermatocytes, round spermatids and elongated spermatids were isolated from the testes of adult WT males, while spermatogonia were extracted from the testes of 7-day-old WT males. These cells were isolated by STA-PUT velocity sedimentation in bovine serum albumin (2% and 4%; BIOSHARP, Hefei, Anhui, China) as described previously^[20]^, and their purity was determined by immunofluorescence staining. Germ cells were isolated with a two-step enzymatic digestion and stained with Hoechst to assess cell viability. Proteins from all cell types were extracted for tandem mass tags (TMT) labeling.

### Fluorescence-activated cell sorting

Testes from 3- and 4-week-old males were de-capsulated and digested in culture medium containing 1 mg/mL collagenase (Invitrogen, Carlsbad, CA, USA) for 5 min, followed by repeated agitation in medium containing 0.25% trypsin (Invitrogen) and 1 mg/mL DNase (Bomeibio, Hefei, Anhui, China) at 37 ℃ for 10 min. Dissociated cells were stained with Hoechst for 30 min and resuspended in 3 mL DMEM (Invitrogen) with 10% fetal bovine serum (Invitrogen) for fluorescence-activated cell sorting (FACS).

### Primary culture of Sertoli and interstitial cells

Adult mice were used for Sertoli and interstitial cell separation. Briefly, testes were decapsulated and incubated in a culture medium containing 1 mg/mL collagenase for 5 min at 37 ℃. Interstitial cells were collected from the supernatant and placed into cell culture. The remaining seminiferous tubules were further dissociated by agitating in a culture medium containing 0.25% trypsin with 1 mg/mL DNase for 10 min at 37 ℃. The resultant cell pellet was collected by centrifugation, resuspended in culture medium and cultured for 24 h. Cells were then treated with hypotonic solution (Invitrogen) and cultured for another 24 h to collect Sertoli cells.

### Proteomic digestion and TMT labeling

Cells were lysed in protein extraction buffer (8 mol/L urea, 75 mmol/L NaCl, 50 mmol/L Tris, pH 8.2, 1% [vol/vol] EDTA-free protease inhibitor, 1 mmol/L NaF, 1 mmol/L β glycerophosphate, 1 mmol/L sodium orthovanadate, 10 mmol/L sodium pyrophosphate). Protein concentration was measured using the Bradford assay (Beyotime, Shanghai, China). Then, proteins were reduced by 5 mmol/L DTT, alkylated in 14 mmol/L iodoacetamide and digested overnight at 37 ℃ with trypsin in a 1 : 100 enzyme-to-substrate ratio^[21]^. After digestion, peptides were desalted by Sep-Pak column from Waters (Milford, MA, USA). TMT labeling was performed according to the manufacturer's protocol. After labeling, all labeled peptide samples were combined, purified, and lyophilized for further proteomic analysis.

### The liquid chromatography tandem mass spectrometry (LC-MS/MS) analysis and data processing

For high-pH reverse phase fractionation, the TMT-labeled peptide mixture was fractionated using ACQUITY UPLC M-Class with XBridge BEH C18 column (300 μm × 150 mm, 1.7 μm; 130Å, Waters). In total, 30 fractions were collected by using a nonadjacent pooling scheme within a 128 min gradient of 3% buffer B for 14 min, 3%–8% B for 1 min, 8%–29% B for 71 min, 29%–41% B for 12 min, 41%–100% B for 1 min, 100% B for 8 min, 100%–3% B for 1 min followed by 20 min at 3% B.

For LC-MS analyses, TMT-labeled peptides were resuspended in 0.1% formic acid and analyzed using an Orbitrap Fusion Lumos mass spectrometer (Thermo Finnigan, San Jose, CA, USA) coupled to the Easy-nLC 1200. Peptides were separated by analytical column (75 μm × 25 cm, Acclaim PepMap RSLC C18 column, 2 μm, 100Å; DIONEX) using a 95 min linear gradient (3%–5% B for 5 s, 5%–15% B for 40 min, 15%–28% B for 34 min and 50 s, 28%–38% B for 12 min, 30%–100% B for 5 s, and 100% B for 8 min).

Raw files were searched against the mouse protein sequences from the Universal Protein Resource (UniProt) database by MaxQuant software (version 1.6.5.0). Carbamidomethyl (C) on cysteine, TMT reagent adducts on lysine and peptide amino termini were set as fixed modifications. Variable modifications included oxidation (M) and acetylation (protein N-term). False discovery rate cut-offs were set to 0.01 for proteins and peptides. The corrected TMT reporter intensities were used for the TMT-based quantification.

### RNA extraction and RT-PCR expression analysis

Mouse tissues were homogenized in TRizol Reagent (Invitrogen), and RNA was extracted following the manufacturer's instructions^[22]^. Total RNA (1 μg) was reversely transcribed by the PrimeScript RT Master Mix (Takara, Beijing, China) and then amplified with the SYBR Green PCR Master Mix (Vazyme, Nanjing, Jiangsu, China) on the Q5 Real-Time PCR System (Applied Biosystems, Waltham, MA, USA). Samples were analyzed in triplicates with *18S* rRNA as the internal control. Gene expression level relative to *18S* rRNA was calculated by the 2^−ΔCt^ method.

### Histology analysis

Hematoxylin and Eosin staining was performed as described previously^[23]^. Briefly, testes were fixed in the modified Davidson's Fluid, paraffin-embedded, and sectioned at 5 µm. Deparaffinized and rehydrated sections were stained with hematoxylin and eosin. Sperm samples obtained from the caud epididymis were spread onto microscope slides, air-dried, and fixed with 4% paraformaldehyde (Sigma, St. Louis, MO, USA). All observations were made using bright field microscopy (Nikon Ni-E, Tokyo, Japan).

### Immunofluorescence staining

Testes were directly embedded in tissue freezing medium, sectioned at 5 µm, and fixed with 4% paraformaldehyde. Slides were blocked with bovine serum (Sigma), and stained with primary antibodies. The primary antibodies used were as follows: anti-PEX16 (1∶200; PA5-60311, Invitrogen), anti-ABCD1 (1∶200; 11159-1-AP, Proteintech, Manchester, UK), anti-ABCD3 (1∶200; PA1-650, Invitrogen), anti-TEX14 (1∶500; ab41733, Abcam, Cambridge, UK), anti-DDX4 (1∶200; ab27591, Abcam), anti-γ-H2AX (1∶200; ab26350, Abcam), anti-SOX9 (1∶200; AB5535, Millipore, Billerica, MA, USA), anti-PLZF (1∶200; AF2944, R&D Systems, Minneapolis, MN, USA). Secondary antibodies were FITC- or TRITC-conjugated goat anti-mouse or anti-rabbit IgG and donkey anti-goat IgG (1∶1000; Beijing Zhongshan Biotechnology Co., Beijing, China). The slides were visualized using a ZEISS LSM800 (ZEISS, Jena, Germany) microscope.

### Terminal deoxynucleotidyl transferase-mediated dUTP-biotin nick end labeling (TUNEL) assay

Testis sections were deparaffinized, permeabilized with proteinase K (20 mg/mL), and stained with the TUNEL BrightRed Apoptosis Detection Kit (Vazyme, Nanjing, Jiangsu, China) according to the manufacturer's instructions. Nuclei were visualized with Hoechst staining. Imaging was performed with a confocal microscope (ZEISS LSM800).

### Ultrastructure analysis

For electron microscopy, testes from 4-week-old mice were fixed in 5% (vol/vol) glutaraldehyde, embedded, sectioned and examined with a TecnaiG2 transmission electron microscope (FEI, Oregon, USA).

### Protein preparation and Western blotting analysis

Protein extracts were prepared using lysis buffer (8 mol/L urea, 75 mmol/L NaCl, 50 mmol/L Tris, pH 8.2, 1% [vol/vol] EDTA-free protease inhibitor, 1 mmol/L NaF, 1 mmol/L β glycerophosphate, 1 mmol/L sodium orthovanadate, 10 mmol/L sodium pyrophosphate, 1 mmol/L PMSF) containing a 1% (wt/vol) protease inhibitor mixture (ThermoScientific, Waltham, MA, USA). The proteins were separated by SDS-PAGE. The membranes were blocked and incubated with indicated primary antibodies. The following primary antibodies were used: anti-PEX16 (1∶1000; PA5-60311, Invitrogen), anti-MRPS22 (1∶1000; 10984-1-AP, Proteintech), anti-PEX14 (1∶1000; 10594-1-AP, Proteintech), anti-GNPAT (1∶1000; ab75060, Abcam), anti-AGPS (1∶1000; ab184186, Abcam), anti-HSD17B4 (1∶1000; 15116-1-AP, Proteintech), anti-β-actin (1∶5000; AC026, ABclonal, Wuhan, Hubei, China). The specific antibody for PEX3 was generated according to the published method^[24]^. Following secondary antibody incubation, detected protein signals were visualized with SuperSignal West Femto Chemiluminescent Substrate (Thermo Scientific) on the Tanon 5200 image system. Intensities were analyzed with Image J and GraphPad Prism.

### ROS detection and lipid peroxidation assay

Testes ROS levels were detected with the ROS fluorescent Probe-Dihydroethidium (DHE) kit (Vigorous, Beijing, China). Briefly, unfixed frozen testis sections were strained with 2 μmol/L DHE in a dark humidified chamber at 37 ℃ for 30 min, followed by Alexa Fluor 555 (Invitrogen) and imaged by fluorescence microscopy (ZEISS LSM800).

Malondialdehyde (MDA) was detected by using the Lipid Peroxidation MDA Assay Kit (Beyotime). The specific experimental procedures are available at http://www.beyotime.com.

### Sperm analysis

Sperm motility and number were measured using a computer-aided sperm analysis (CASA) system as described previously^[23]^. Sperm from sexually mature mice were isolated from the cauda epididymis by placing the incised tissue in 0.05, 0.20, or 0.50 mL of mHTF (Irvine Scientific, Santa Ana, CA, USA) for 5 min at 37 ℃. Cell debris was removed and an aliquot of sperm was analyzed on the IVOS Sperm Analyzer (Hamilton Torne Biosciences, Beverly, MA, USA).

### Statistical analysis

Statistical analyses were conducted by the GraphPad Prism 7.0 software (GraphPad, USA). Two-tailed unpaired Student's *t*-tests were used for two-group comparisons. Data were presented as mean ± standard error of the mean. *P* < 0.05 is defined as statically significant.

## Results

### Heterogeneity of peroxisomes among organs as well as between somatic and spermatogenic cells in testis

**Figure 1:**
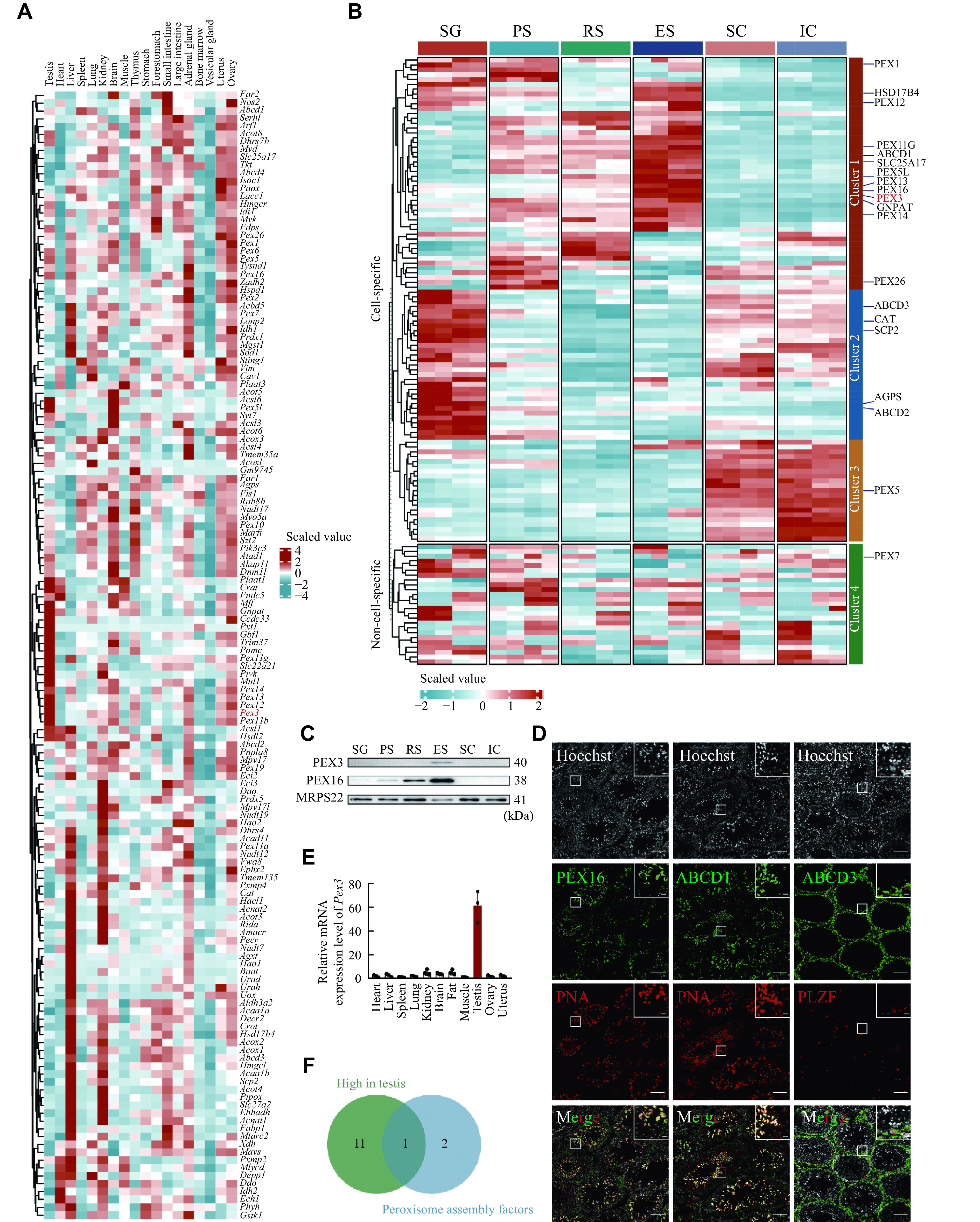
Heterogeneity of peroxisomes in organs and testicular cells.

To examine the expressions of PMPs and peroxisomal enzymes in various organs, we analyzed previous single-cell RNA sequencing data of mouse testis, heart, liver, spleen, lung, kidney, brain, muscle, thymus, stomach, forestomach, small intestine and large intestine, adrenal gland, bone marrow, vesicular gland, uterus, and ovary^[25]^. We revealed that the expressions of peroxisomal genes were largely heterogeneous among different organs, with approximately half of them being enriched in the testis, liver, or kidney. For example, *Abcd3*, a PMP involved in the transportation of long-chain acyl-CoA across the peroxisomal membrane, and catalase, a peroxisomal enzyme for defending against oxidative stress, were both highly expressed in the liver and kidney (***Fig. 1A***). Interestingly, we noticed that a population of peroxisomal genes was highly expressed in the testis, including *Gnpat*, the gene coding for a key enzyme played a crucial role in the initial step of ether lipids synthesis, and the genes coding for peroxisomes assembly factors, such as *Pex3*, *Pex13*, and *Pex14* (***Fig. 1A***). The fact that these genes were abundantly expressed in the testis indicated that they likely had important regulatory functions in spermatogenesis.

The testis is known to contain a variety of somatic and germ cell types. To further detect the distribution of PMPs and peroxisomal enzymes, we performed the TMT-based quantitative proteomic analysis of testicular cells, including spermatogonia, spermatocytes, round spermatids and elongated spermatids, Sertoli cells and interstitial cell populations (***Supplementary Fig. 1A*** and ***1B***, available online). The expression levels of peroxisomal proteins displayed a remarkable heterogeneity between germ and somatic cells, also among the four different developmental stages of germ cells (***Fig. 1B***). For instance, the cluster 2 peroxisomal proteins, such as ABCD3 and catalase, were highly expressed in spermatogonia with roughly half of the proteins exhibiting mild expression in somatic cells (***Fig. 1B***). Over half of the peroxisomal proteins in cluster 1 showed a gradual increase in expression from spermatocytes to spermatids. The cluster 1 peroxisomal proteins, GNPAT and PEX3, were expressed at low levels in pre-meiotic germ cells but highly in round spermatids and elongated spermatids (***Fig. 1B***). To validate the proteomic results, we performed Western blotting analyses of PEX3 and PEX16 in the isolated germ cells and somatic cells. The results indicated that PEX3 and PEX16 were most highly expressed in elongated spermatids, which was consistent with the proteomic data (***Fig. 1C***). The localization of PEX16 and ABCD1 with PNA, a marker of spermatid acrosomes, and ABCD3 with PLZF, a marker of spermatogonia in adult testes, showed that both PEX16 and ABCD1 were highly expressed in spermatids, whereas ABCD3 was highly expressed in PLZF-positive spermatogonia (***Fig. 1D***). In addition, PMPs and peroxisomal enzymes exhibited heterogeneous expressions between germ cells and somatic cells, as well as across different developmental stages of germ cells, indicating possible heterogeneous functions of peroxisomes in germ and somatic cells.

### Deletion of *Pex3* specifically in germ cells caused spermatogenic failure

**Figure 2:**
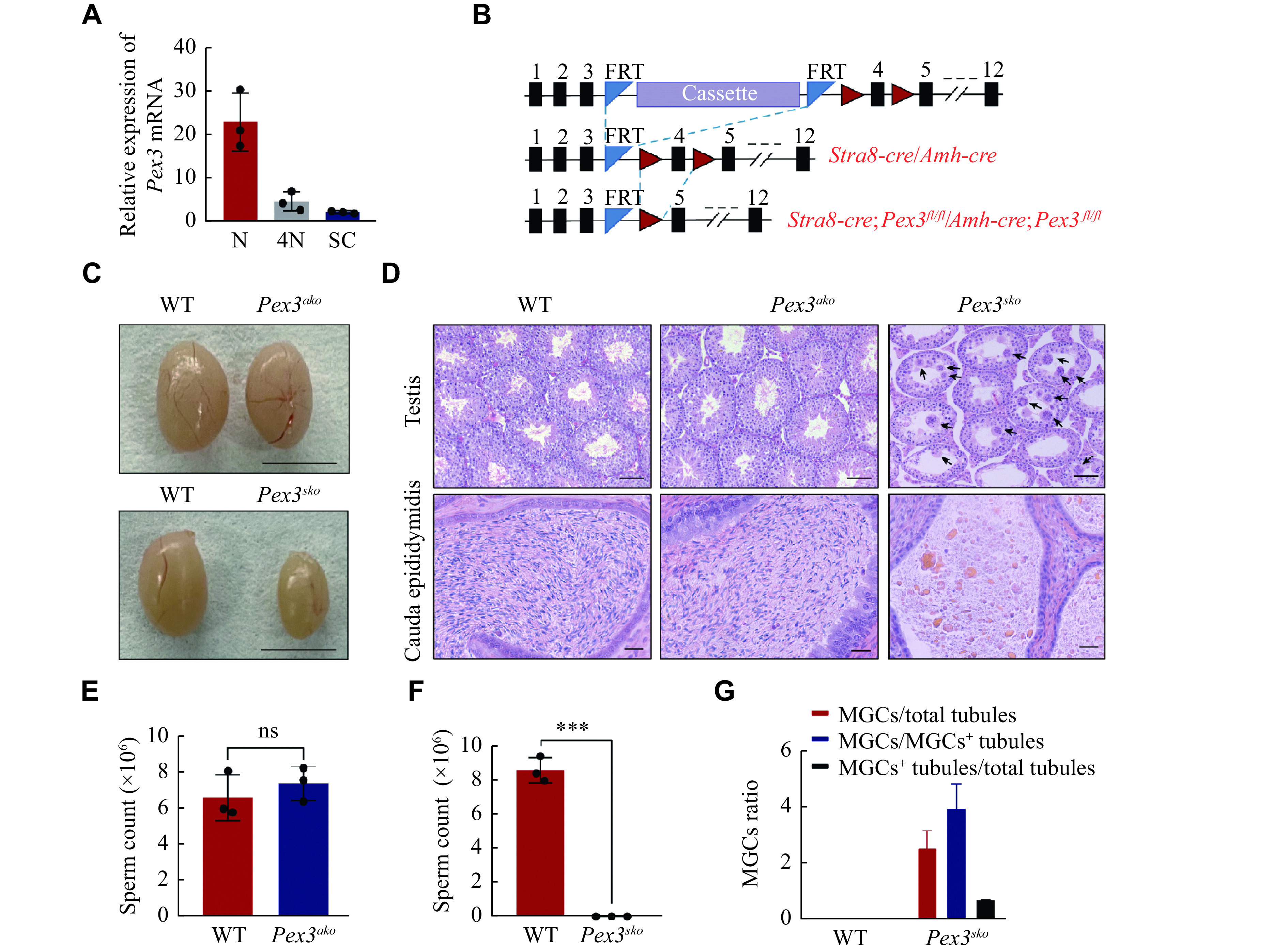
Specific deletion of *Pex3* in germ cells triggered spermatogenic failure.

The peroxisome biogenesis factor, *Pex3*, was testis-abundant and highly expressed in spermatids (***Fig. 1E*** and ***1F***), contributing to male fertility as reported by Zhao *et al*^[13]^. Since *Pex3* was expressed in both germ and Sertoli cells (***Fig. 2A***), it is important to investigate in which cell type the PEX3-dependent peroxisomes play a role during spermatogenesis. We generated *Pex3* floxed (*Pex3*^*fl/fl*^) mice and crossed them with the *Stra8*-*Cre* or *Amh*-*Cre* transgenic mice to generate conditional knockout of *Pex3* in germ cells or Sertoli cells, respectively (***Fig. 2B***). We isolated germ cells from *Stra8*-*Cre*;*Pex3*^*fl/fl*^ (*Pex3^sko^*) and WT testes, and cultured Sertoli cells from *Amh*-*Cre*;*Pex3*^*fl/fl*^ (*Pex3^ako^*) and WT testes to verify *Pex3* knockout efficiency. Both *Pex3*^*sko*^ germ and *Pex3*^*ako*^ Sertoli cells showed a significant reduction in *Pex3* mRNA (***Supplementary Fig. 1C*** and ***1D***, available online). Morphological analysis showed that *Pex3*^*ako*^ mice had a normal testis size and spermatogenesis in the testes and epididymis (***Fig. 2C*** and ***2D***,*** Supplementary Fig. 1E*** [available online]). Sperm counts and motility were also unaltered, and *Pex3*^*ako*^ males were able to reproduce with WT females (***Fig. 2E***, ***Supplementary Fig. 1F***–***1I*** [available online]). In contrast, *Pex3*^*sko*^ mice exhibited a reduced testis size and male infertility (***Fig. 2C*** and ***2D***, ***Supplementary Fig. 1J*** and ***1K*** [available online]). Histological analyses showed the arrested spermatogenesis at the round spermatids stage in most seminiferous tubules, with the formation of multinucleated cells containing up to 30 nuclei, and an increasing number of vacuoles in the lumens (***Fig. 2D*** and ***2G***). Additionally, the epididymis of *Pex3*^*sko*^ mice was filled with cell debris instead of spermatozoa (***Fig. 2D*** and ***2F***). Therefore, PEX3-dependent peroxisomes were dispensable for Sertoli cells but essential for normal spermatogenesis in germ cells.

### MGCs began in *Pex3*^*sko*^ testis when spermatids appeared

**Figure 3:**
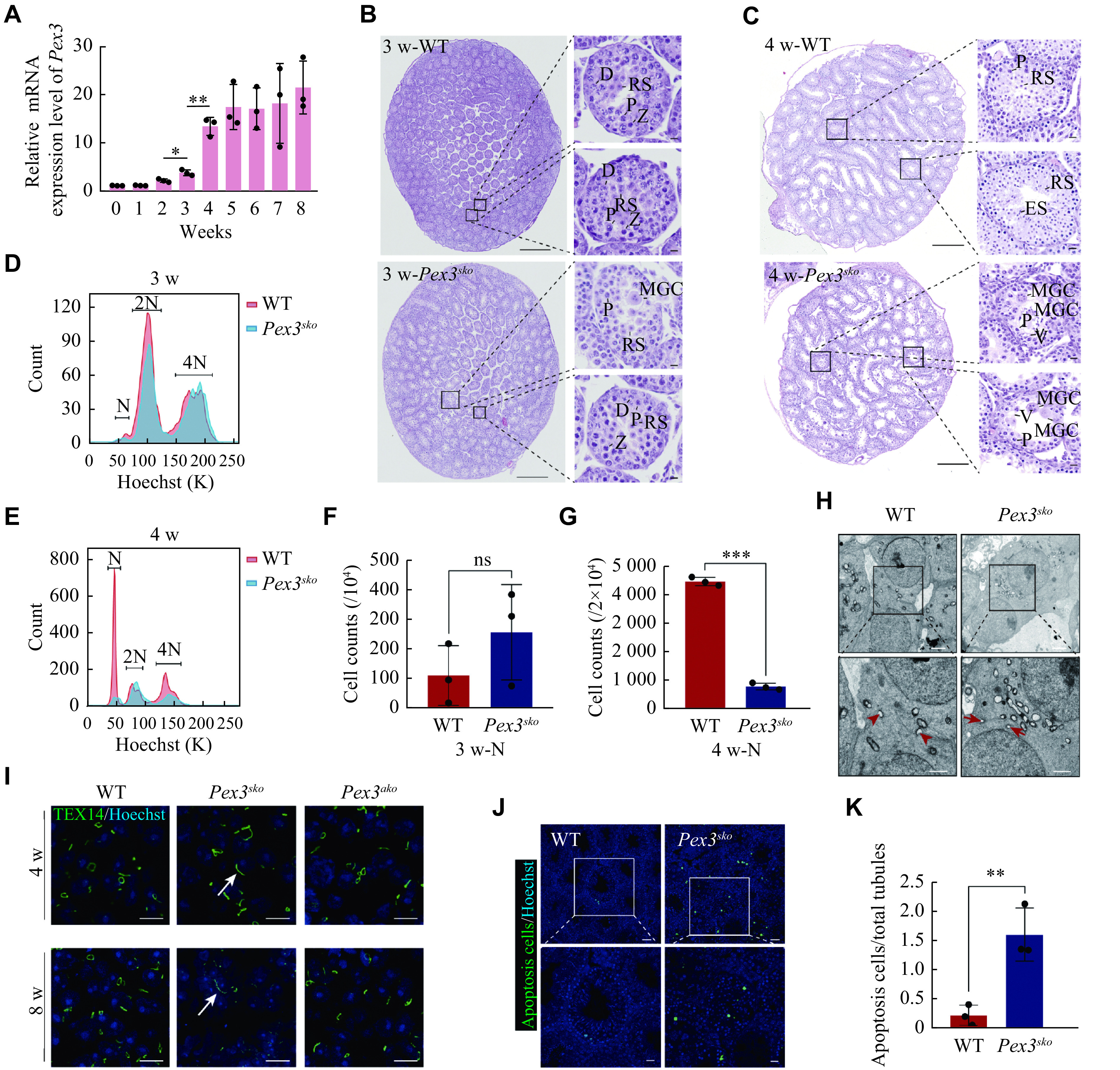
MGCs occur when spermatids develop.

Proteomic findings showed that PEX3 was highly expressed in round spermatids and peaked in elongated spermatids. Consistently, we observed a significant increase in *Pex3* mRNA expression from 3- to 4-week, when spermatids appeared^[26]^ (***Fig. 3A***). We confirmed that MGCs were present from week 3 in *Pex3*^*sko*^ testes and further increased by week 4 (***Fig. 3B*** and ***3C***). Further FACS analysis of 3- and 4-week-old *Pex3*^*sko*^ testes revealed that the number of haploid cells did not significantly change in 3-week but decreased in 4-week *Pex3*^*sko*^ testes (***Fig. 3D***–***3G***). This illustrated that PEX3-dependent peroxisomes affected spermatids development in the first wave of spermatogenesis of *Pex3*^*sko*^ testes. As the deficiency of intercellular bridges (ICBs) leads to the formation of MGCs^[27]^, we examined the formation of ICBs through transmission electron microscopy. *Pex3*^*sko*^ testes showed a decreased dense material beneath the plasma membrane of ICBs, leading to the broadening of the ICBs and the formation of MGCs in the tubules (***Fig. 3H***). Immunostaining of TEX14, a marker of ICBs, showed an increased length of ICBs in *Pex3*^*sko*^ testes at 4- and 8-weeks, but not in *Pex3*^*ako*^ testes (***Fig. 3I***). Additionally, cell apoptosis dramatically increased in *Pex3*^*sko*^ testes (***Fig. 3J*** and ***3K***). The above results suggest that PEX3-dependent peroxisomes play a role in the process of spermiogenesis, the absence of which leads to the destruction of ICBs, and the subsequent formation of massive MGCs is composed of spermatids.

### *Pex3* deficiency affected peroxisomal proteins in spermatids

**Figure 4:**
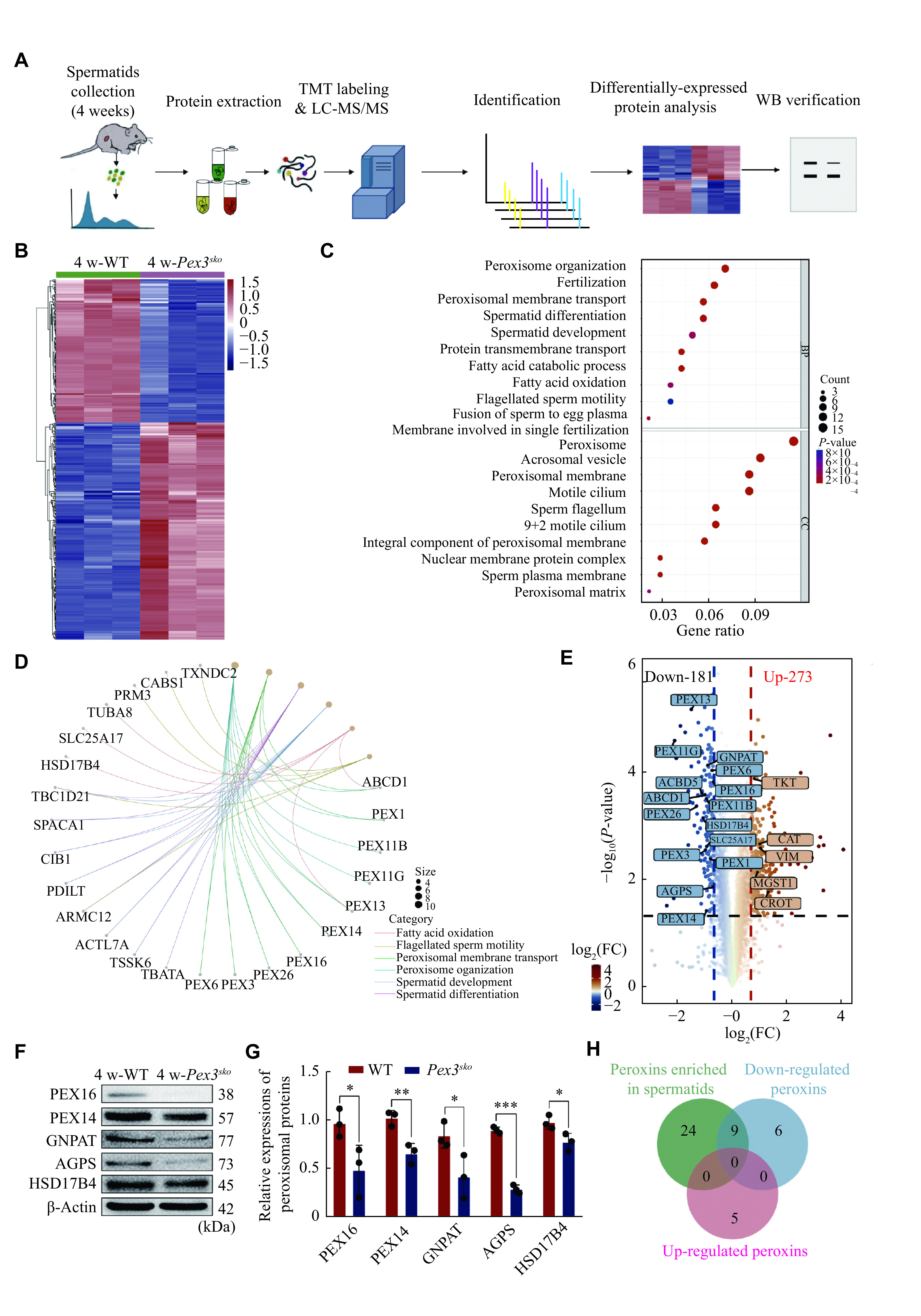
Deletion of *Pex3* triggers differentially regulated proteins involved in spermatids.

To evaluate the protein changes resulting from *Pex3* deletion in spermatids, we purified haploid spermatids from 4-week-old WT and *Pex3*^*sko*^ testes with FACS (***Fig. 4A***), followed by the TMT-based mass spectrometry analysis. *Pex3*^*sko*^ spermatids displayed 273 up-regulated proteins and 181 down-regulated proteins (***Fig. 4B***). Gene Ontology enrichment analysis and the gene network showed the enrichment of down-regulated proteins in peroxisome biogenesis, including PEX3 and PEX16, in spermatid formation, including TSSK6 and PRM3, and in lipid metabolism, such as HSD17B4 and SLC25A17 (***Fig. 4C*** and ***4D***). Moreover, the GSEA analysis of KEGG pathway enrichment showed a lower abundance of peroxisomal-related proteins in *Pex3*^*sko*^ spermatids (***Supplementary Fig. 2A***, available online), while up-regulated proteins were associated with the maintenance and homeostasis of organelles (***Supplementary Fig. 2B*** and ***2C***, available online). Given that *Pex3* is responsible for the biogenesis of peroxisomes, the disruption of *Pex3* is related to the absence of peroxisomes^[28]^. We further analyzed the changes of peroxisomal proteins in *Pex3*^*sko*^ spermatids. Among the differentially expressed proteins, 15 peroxisomal proteins were down-regulated and five were up-regulated in *Pex3*^*sko*^ spermatids (***Fig. 4E***). Eight of the down-regulated proteins, including PEX13, PEX14, PEX16, GNPAT, ABCD1, peroxisomal biogenesis factor 11 gamma (PEX11G), solute carrier family 25 member 17 (SLC25A17), and hydroxysteroid 17-beta dehydrogenase 4 (HSD17B4), are enriched in spermatids, consistent with PEX3 in Cluster 1 (***Fig. 1B***, ***Fig. 4E*** and ***4H***). To verify the proteomics data by LC-MS/MS, five peroxisomal proteins that decreased in 4-week-old *Pex3*^*sko*^ spermatids were selected, including two proteins involved in biogenesis of peroxisome, PEX16 and PEX14, and two proteins related to ether lipid synthesis, GNPAT and alkylglycerone phosphate synthase (AGPS), and one protein associated with β-oxidation of very-long-chain fatty acids, HSD17B4. Western blotting results confirmed the lower expression of these proteins in *Pex3*^*sko*^ testes at 4-week (***Fig. 4F*** and ***4G***). This suggests that the eight down-regulated proteins may be in the same peroxisomes with PEX3, and the loss of PEX3-dependent peroxisomes may affect the normal expression of proteins related to spermatid formation.

### Germ cell-specific deletion of PEX3-dependent peroxisomes led to the increased oxidative stress in testes

**Figure 5:**
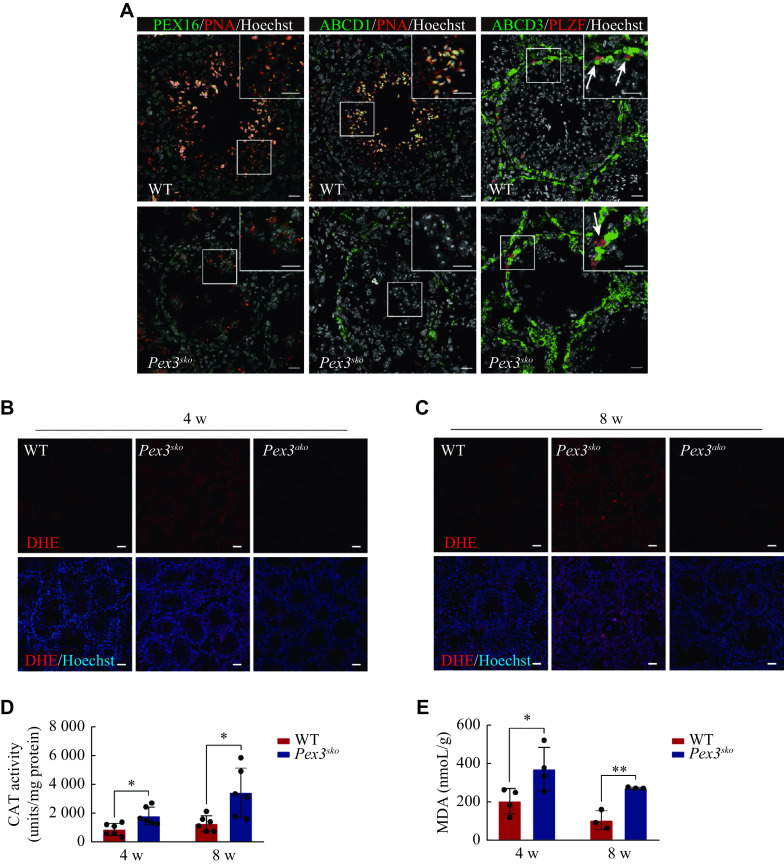
Deficiency of PEX3-dependent peroxisomes leads to the increased oxidative stress in the testes.

Previous studies showed that *Pex3* mutation in mammals resulted in the abnormal distributions of PMPs and led to failures in peroxisomal proteins transportation and membrane vesicle assembly^[10]^. We performed immunofluorescence staining analysis of two typical PMPs, PEX16 and ABCD1, and observed a decreased expression of PEX16 and the absence of ABCD1 in *Pex3*^*sko*^ spermatids (***Fig. 5A***). However, the expression and location of ABCD3, a PMP mainly enriched in spermatogonia and somatic cells, were not affected by the *Pex3* deletion in germ cells. The consistent expression pattern and downregulation of PEX14, PEX16, and ABCD1 after the *Pex3* deletion suggested that these proteins were located in the PEX3-dependent peroxisomes, while ABCD3 might not be in the PEX3-dependent peroxisomes. Peroxisomes are essential for the removal of excess ROS, and improper management may lead to lipid peroxidation within cells^[29]^. To evaluate whether ROS clearance defects were present in the two conditional knockout mice, we used a DHE probe to measure ROS levels. As expected, we observed significantly higher ROS levels in the testes of 4- and 8-week-old *Pex3*^*sko*^ mice but not in *Pex3*^*ako*^ mice (***Fig. 5B*** and ***5C***). Catalase was an enzyme involved in peroxisomal clearance of ROS^[30]^, and its activities were increased in *Pex3*^*sko*^ testes at 4- and 8-weeks (***Fig. 5D***). Lipid peroxidation, as measured by the level of MDA, was also elevated in the 4- to 8-week-old *Pex3*^*sko*^ testes (***Fig. 5E***). These results indicate that PEX3-dependent peroxisomes mainly function in the development of spermatids, and their defects lead to increased levels of ROS and lipid peroxidation, eventually blocking the formation of mature spermatids.

## Discussion

Several reports have previously suggested the existence of functional heterogeneities in peroxisomes in different organs^[8,31]^. However, the composition heterogeneity of peroxisomes and the molecular basis for these functional differences are still not well understood. In the current study, we found that expressions of peroxisomal proteins were heterogeneous among organs and during the developmental stages of spermatogenesis in mice. In particular, the germ cell- but not Sertoli cell-specific deletion of a testis-preferential peroxisomal protein, PEX3, led to the absence of PEX3-dependent peroxisomes in spermatids and increased levels of ROS in germ cell-specific knockout testes, which resulted in the formation of MGCs composed of spermatids, defects in spermiogenesis, and eventually male infertility.

Previously, Zhao *et al*^[13]^ conducted a study on oxidative stress by knocking out *Pex3* and showed infertility in *Pex3*^−*/*−^ male mice. However, the specific testicular cell types in which *Pex3* functions remain unknown, while *Pex3* is expressed in both germ and Sertoli cells. Proteomic analysis showed heterogeneous expression of peroxisomal proteins across different cell types of testes, especially between germ and Sertoli cells. Our conditional knockout of *Pex3* in germ and Sertoli cells has shown that while *Pex3* is necessary for spermatogenesis in germ cells, it is not critical in Sertoli cells. Unlike defects in other *Pex* genes, *Pex3* and *Pex19* mutants do not produce peroxisomal membrane remnants (ghosts)^[32]^, but we found that germ cell-specific knockout of *Pex3* led to a dramatic reduction in other spermatid-abundant peroxisomal proteins, such as PEX14, PEX16, ABCD1, GNPAT, and HSD17B4. Previous studies also showed that the inactivation of GNPAT in the testes induced MGCs formation in the seminiferous tubules at the spermatids stage^[12]^. These proteins, such as PEX14, PEX16, ABCD1, GNPAT, and HSD17B4, may exist in PEX3-dependent peroxisomes; however, further experimental studies are still needed to confirm these results.

During the spermatid stage, peroxisomes help to remove excess ROS and synthesize ether lipids to maintain synchronous maturation of spermatogenic cells^[33]^. We observed a massive increase in the number of MGCs in the testes of 4-week-old *Pex3*^*sko*^mice, along with the elevated ROS levels, suggesting a correlation between oxidative stress and MGC production. In germ cells, ICBs are synthesized by altering the fate of the midbody, thus connecting germ cells derived from the same spermatogonium in a syncytium^[34–35]^. Lipids, such as sphingolipids containing ultra-long polyunsaturated acids, are critical for stabilizing the ICBs of spermatids^[27]^. Disruptions in ether lipid synthesis can cause the breakdown of ICBs and lead to the generation of MGCs^[16]^. Because the current study found the decreased expression of GNPAT and AGPS, two proteins related to ether lipid synthesis, we speculate that the production of MGCs is also related to the pathway of ether lipid synthesis, although the mechanism remains to be defined.

In summary, the current study has demonstrated that PEX3-dependent peroxisomes in germ cells are indispensable for spermatogenesis, especially at the stage of spermatids. Defects in these peroxisomes are accompanied with the elevated levels of ROS. However, we are currently unaware of the specific peroxisomal proteins in PEX3-dependent peroxisomes, and it is unclear whether other types of peroxisomes exist. Furthermore, the functions of heterogeneous peroxisomes in other testicular cells remain to be elucidated.

## SUPPLEMENTARY DATA

Supplementary data to this article can be found online.
