# Supplementary material for: Germ cell-specific deletion of Pex3 reveals essential roles of PEX3-dependent peroxisomes in spermiogenesis
Source: J Biomed Res. 2023 Dec 8;38(1):24–36. doi: 10.7555/JBR.37.20230055 (PMC10818173; doi:10.7555/JBR.37.20230055)
Supplement: Supplementary file 1 — Supplementary data to this article can be found online. [file jbr-38-1-24-S1.pdf]

Available online at www.jbr-pub.org.cn

Open Access at PubMed Central

The Journal of Biomedical Research, 2023 37(0): 1–2

JBR

*Original Article*

# Germ cell-specific deletion of *Pex3* reveals essential roles of PEX3-dependent peroxisomes in spermiogenesis

Yejin Yao[1,△], Baolu Shi[2,△], Xiangzheng Zhang[1,△], Xin Wang[1], Shuangyue Li[1], Ying Yao[1], Yueshuai Guo[1], Dingdong Chen[1], Bing Wang[1], Yan Yuan[1], Jiahao Sha[3,✉], Xuejiang Guo[1,✉]

[1]*State Key Laboratory of Reproductive Medicine and Offspring Health, Nanjing Medical University, Nanjing, Jiangsu 211166, China;*

[2]*Reproductive and Genetic Branch, the First Affiliated Hospital of USTC, Division of Life Sciences and Medicine, University of Science and Technology of China, Hefei, Anhui 230001, China;*

[3]*State Key Laboratory of Reproductive Medicine and Offspring Health, Women's Hospital of Nanjing Medical University, Nanjing Maternity and Child Health Care Hospital, Nanjing Medical University, Nanjing, Jiangsu 211166, China.*

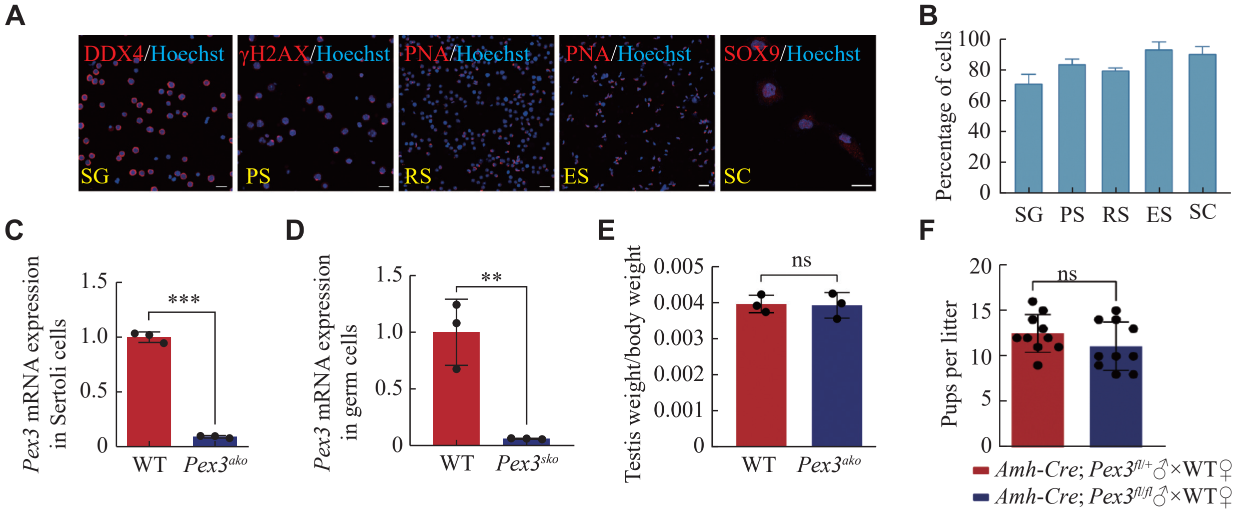


(Continued)

△These authors contributed equally to this work.

✉Corresponding authors: Jiahao Sha, State Key Laboratory of Reproductive Medicine and Offspring Health, Women's Hospital of Nanjing Medical University, Nanjing Maternity and Child Health Care Hospital, Nanjing Medical University, 101 Longmian Road, Nanjing, Jiangsu 211166, China. Tel/Fax: +86-25-86869387. E-mail: shajh@njmu.edu.cn; Xuejiang Guo, State Key Laboratory of Reproductive Medicine and Offspring Health, Nanjing Medical University, 101 Longmian Road, Nanjing, Jiangsu 211166, China. Tel/Fax: +86-25-86869383. E-mail: guo_xuejiang@njmu.edu.cn.

Received: 17 March 2023; Revised: 29 May 2023; Accepted: 29 May 2023; Published online: 08 December 2023

CLC number: R321.1, Document code: A

The authors reported no conflict of interests.

This is an open access article under the Creative Commons Attribution (CC BY 4.0) license, which permits others to distribute, remix, adapt and build upon this work, for commercial use, provided the original work is properly cited.

© 2023 by the Journal of Biomedical Research.

https://doi.org/10.7555/JBR.37.20230055

(Continued)

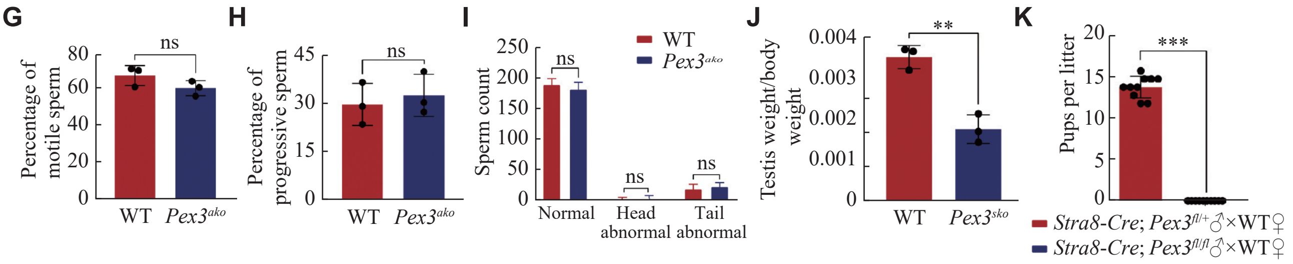


***Supplementary Fig. 1*** **Deletion of *Pex3* specifically in germ cell not Sertoli cell led to male infertility.** A: Immunostaining revealed the purity of freshly isolated spermatogonia (SG), pachytene spermatocytes (PS), round spermatids (RS), elongated spermatids (ES) and Sertoli cells (SC). Cell nuclei were stained with Hoechst (blue). DDX4, germ cells. γ-H2AX, DNA double strand breaks. PNA, acrosome of round and elongated spermatids. SOX9: Sertoli cells. Scale bars, 20μm. B: Quantification of purity of testicular cells, SG: 71.8%; PS: 84.4%; RS: 80.5%; ES: 94.1%; SC: 91.1%. Two biological replicates for each cell type. C and D: Real-time PCR quantification of *Pex3* mRNA expression in Sertoli cells and germ cells from $Pex3^{ako}$, $Pex3^{sko}$, and control males. $n$ = 3. To better show knockout efficiency, the relative gene expression values were normalized to the WT level. E: Testis-to-body weight ratio in WT (0.00398) and $Pex3^{ako}$ (0.00395) mice. $n$ = 3. F: The average number of pups per litter between adult control and $Pex3^{ako}$ males. $n$ = 10. G: Percentage of motile sperm from WT and $Pex3^{ako}$ males. $n$ = 3. H: Percentage of progressive sperm from WT and $Pex3^{ako}$ males. $n$ = 3. I: Counts of sperm exhibiting abnormal morphological characteristics in WT and $Pex3^{ako}$ mice. J: Testis-to-body weight ratio in WT (0.00349) and $Pex3^{sko}$ (0.00173) mice. $n$ = 3. K: The average number of pups per litter between adult control and $Pex3^{sko}$ males. $n$ = 10. Three pair mice were counted. At least two hundred sperm were counted for each mouse. Data are presented as mean ± standard error of mean (SEM). Two-tailed unpaired Student's $t$-test was applied for two-group comparisons. $^{**}P < 0.01$ and $^{***}P < 0.001$. Abbreviation: ns, not significant.

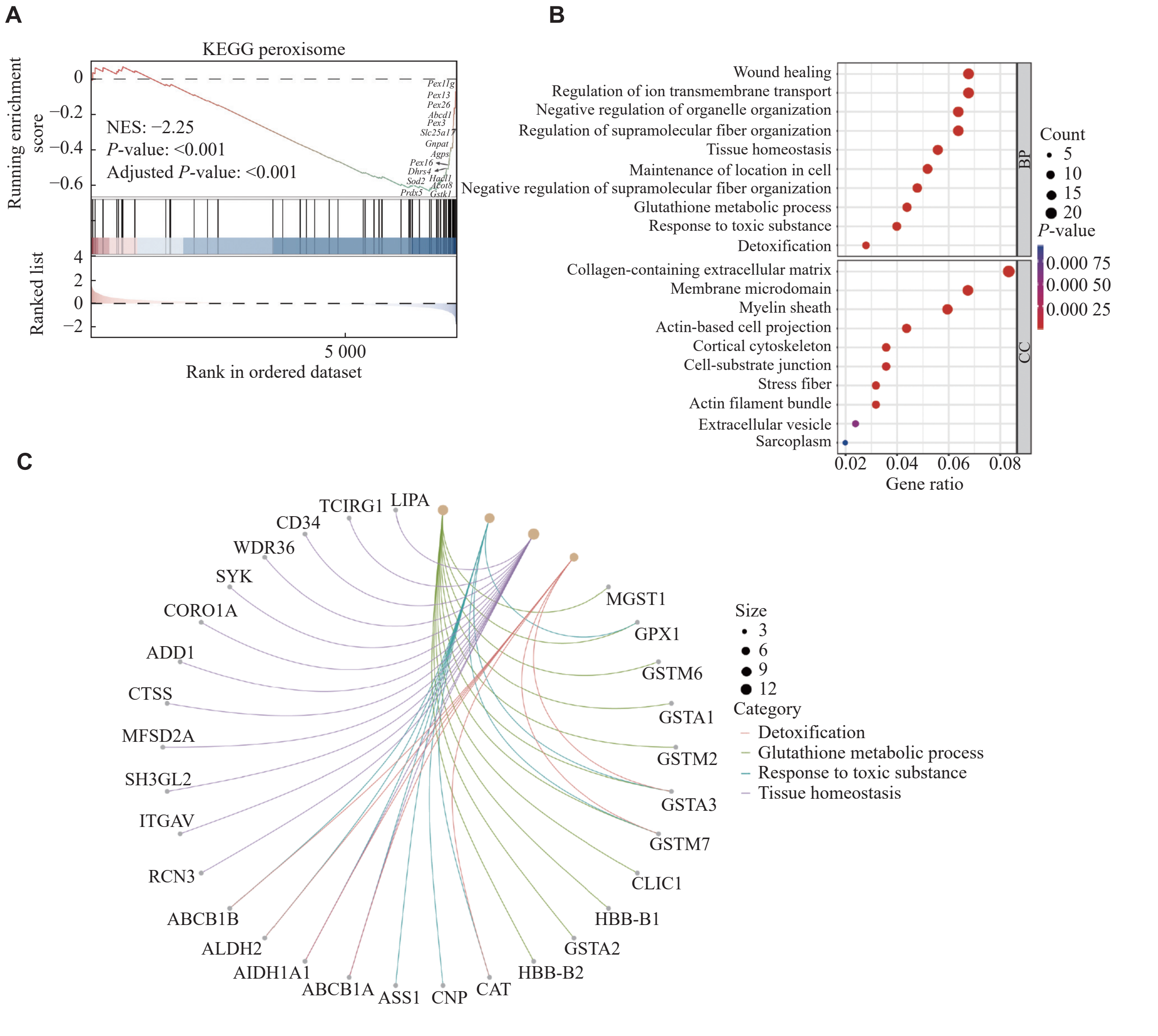


***Supplementary Fig. 2*** **Biological analysis of differential proteins between 4-week-old WT and $Pex3^{sko}$ spermatids.** A: GSEA analysis of peroxisomal pathway-related proteins between 4-week-old WT and $Pex3^{sko}$ spermatids. Proteomics data was used here. B: Gene Ontology enrichment analysis of upregulated peroxisomal proteins. C: The network of the linkages of genes and biological events of upregulated peroxisomal proteins in (B).
